# First-Hand Accounts of Suicidal Mental Imagery: A Taxonomy of Imagery Types

**DOI:** 10.3390/bs16060908

**Published:** 2026-06-03

**Authors:** Marie Carey, Brian Keogh, Louise Doyle

**Affiliations:** 1 Department of Nursing and Healthcare, South East Technological University, X91 K0EK Waterford, Ireland; 2 School of Nursing and Midwifery, Trinity College Dublin, 24 D’Olier Street, D02 T283 Dublin, Ireland; keoghbj@tcd.ie (B.K.); doylel1@tcd.ie (L.D.)

**Keywords:** assessment and treatment, CBT suicide prevention (CBT-SP), cognitive behavioural therapy (CBT), comfort, distress, suicidal mental imagery (SUMI), suicide prevention, taxonomy

## Abstract

Suicidal mental imagery are self-generated images of injury and death experienced across a range of mental health conditions, but also experienced in people who have no diagnosable mental health condition. For some, these images can be quite distressing and unwanted, but for others, these mental pictures serve to bring comfort and problem-solving capabilities. From a clinical and research perspective, they are not fully understood and are underexplored. Suicidal mental imagery is a known risk factor for attempted suicide due to imagery’s amplification and habituation effects, potentially moving a person from imagining suicide to acting on it. This paper presents a taxonomy of the different types of suicidal mental imagery from first-hand accounts extracted from phase one of a two-phase qualitative study called the suicidal mental imagery (SUMI) study. This taxonomy of imagery types is drawn from fifteen semi-structured interviews exploring imagery experiences in adults from Ireland. The characteristics of these images were analysed qualitatively using an Interpretive Description approach, with the resulting taxonomy developed through additional analytic processes informed by cognitive behavioural therapy (CBT) principles and a priori coding. The taxonomy, therefore, illustrates experiences from a rich dataset under distinct categories to improve our understanding of the key mechanisms underpinning suicidal mental imagery for practice, helping to assist open dialogue in imagery assessment to better inform suicide prevention interventions.

## 1. Introduction

Suicidal ideation is not just streams of ‘verbal thoughts’ or mental planning; it is also made up of mental pictures (Holmes et al., 2007). Suicidal mental imagery occurs when a person sees a picture of self-injury or death in the mind’s eye, as if imagining in the future what ending life would look like or mean to the person. Though there is no agreed or universally accepted definition of suicidal ideation (Harmer et al., 2023), definitions do not routinely include the phenomenon of suicidal mental imagery as a feature of suicidal ideation despite its presence. Suicidal ideation and planning are therefore clinically assessed by seeking out the presence of verbal thoughts rather than by specifically asking about associated mental images or pictures (Schultebraucks et al., 2020). Suicidal mental imagery is a risk factor in moving a person from ideation to action, and there is greater presence of suicidal mental imagery when a person has experienced suicidal ideation previously (Holmes et al., 2007). It is known that there is increased risk of suicidal behaviour when experiencing suicidal mental imagery and it is common among those at risk of suicide (Crane et al., 2012; Hales et al., 2011; Holaday & Brausch, 2015; Holmes et al., 2007; Ng et al., 2016). Although considered a novel, under-researched marker for suicide risk (Lawrence et al., 2022), preliminary evidence from meta-analysis and systematic review of studies from 1998 to 2024 shows that it is linked to suicidal behaviour (Lawrence et al., 2023). Essentially, while clinicians ask about suicidal thoughts and plans during routine mental health assessments—suicidal mental imagery is not routinely captured.

This manuscript aims to illustrate a taxonomy of suicidal mental imagery types, drawing on first-hand accounts collected in phase one of a two-phase study, and applying CBT-informed a priori coding to enhance understanding for clinical practice. The second phase of the SUMI study seeks to explore international expert views on developing pathways for introducing imagery into routine mental health assessment and intervention (phase two is not reported in this paper; its findings will be presented in a future publication). Qualitative research on real-life suicidal experiences remains limited. Heesen et al. (2024) found that half of participants mentally rehearsed a method or scenario, while Nilsson et al. (2023) highlight the lack of understanding of how mental imagery functions within acute suicidal episodes across cognitive, emotional, behavioural, and physiological systems, central to CBT, a robust and well-established evidence base that underpins this taxonomy. This taxonomy may help our understanding of what it means to have injury and death-based imagery across the suicide spectrum, from acute to enduring imagery experiences.

Imagery (as a symptom of the suicidal mind) is not universally known about in routine mental health practice, with Paulik et al. (2023) putting forward an urgent call for research into suicidal imagery interventions. Carey et al. (2024) state that we need to first start asking about SUMI in clinical settings as part of routine mental health assessment to normalise its existence. The limited research on suicidal mental imagery suggests it is associated with increased risk for suicidal behaviour (Crane et al., 2012; Schultebraucks et al., 2020) and has been identified as a volitional motivator (O’Connor & Kirtley, 2018), highlighting that current suicide risk assessment measures may be improved by assessing for suicidal mental imagery. However, capturing the manifestations of suicidal thinking and associated imagery is considered challenging research as it requires people to volunteer their presence, with up to 60% of people not disclosing (Hallford et al., 2023).

It is posited that if clinicians are not assessing suicidal ideation fully (to include imagery), they are not explicitly asking about the presence of death or injury-based imagery during mental health assessment, a potential missed opportunity to stabilise risk. As mentioned, understanding the mechanisms in suicidal thinking is important in our understanding of treatment and stabilisation efforts. CBT-SP (suicide prevention) uses guided imagery exercises in relapse management planning (Stanley et al., 2009; Wenzel & Jager-Hyman, 2012). This CBT manualised treatment for suicide, could be further innovated to expressly include imagery interventions as a central target for treatment in the earlier more acute phase of suicidality; such as imagery rescripting, to identify suicidal mental imagery and modify this imagery (as the central task to effect change in reducing suicidal risk) by deglamourizing the problem-solving loop of suicidal thinking and by increasing hope through imagined life goals (Carey & Wells, 2019). Engaging front-line clinicians to routinely ask about suicidal mental imagery as part of routine mental health assessment is a priority when we know of (a) the amplification mechanism and (b) the imagery modification potential. For example, clinicians could ask What images run through your mind when you feel like life is not worth living anymore? ‘(Di Simplicio et al., 2012). Carey et al. (2024) suggests that understanding the comforting aspect of suicidal mental imagery is therefore crucial. Clinicians may miss the risk implications of comfort-based suicidal imagery—perceived by the suicidal person as soothing, a person may engage in it to help cope with stress or problem-solve life’s pain, and potentially move from ideation to action. Crane et al. (2014) found that comfort was associated with several markers of a more severe clinical profile, including both worst-ever prior suicidal ideation and worst suicidal ideation over a 12-month follow-up period.

Evidence shows that imagery is assessed far more routinely where there is a diagnosis traditionally associated with having more explicit imagery-based symptomatology (D. G. Pearson et al., 2013). Examples of these disorders include post-traumatic stress disorder (PTSD), concerned with distressing ‘flashbacks’ or obsessive compulsive disorder (OCD), where there might be disgust emotions associated with contamination-based imagery (Hales et al., 2011). For example, the Yale Brown Obsessive Compulsive Scale (YBOC) by Goodman et al. (1989) specifically enquires about such images, serving to prompt more diagnostic accuracy and treatment in OCD. Imagery-based questionnaires are often employed by clinicians or researchers in response to a respondent’s likely diagnosis rather than as part of a routine clinical interview (Hales et al., 2011), for example, the Structured Clinical Interview for DSM-5 (SCID-5), where there is no diagnostic expectation at the outset. Consequently, the SUMI study from which this taxonomy is derived more broadly aims to increase awareness of the importance of enquiring about suicide-related mental imagery within routine or general mental health assessments, particularly where a formal clinical diagnosis may be absent or not applicable. Though suicidal imagery measurement instruments exist, such as the suicidal intrusion attributes scale (SINAS) developed by van Bentum et al. (2023) to assess for compelling suicidal imagery, such scales are not routinely used in practice, remaining an overlooked aspect of suicidal assessment.

Frontline and secondary care mental healthcare clinicians do not receive suicidal mental imagery assessment and intervention training in Ireland, resulting in a training gap in the application of imagery science to practice. The suicidal person presenting to Primary Care or the Emergency Department who may or may not have a diagnosable mental illness is not being assessed for imagery-based risk by the nurse or doctor; key disciplines are largely unaware of the need to include suicidal mental imagery assessment. As mental health science research and imagery-based protocols develop, such clinical interventions may prioritise imagery modification as the central task for clinicians, alongside robust safety planning. Hotopt et al. (2020) examined the COVID-19 pandemic, observing that such mental health science and research need to understand the psychological mechanisms that account for changes in anxiety, depression, self-harm and suicide more generally in the population than, for example, in the constraints of a formal psychiatric diagnosis. They advise that a coordinated research effort is required, meaning that no one discipline will be able to provide interventions to meet the demand and that interventions need to be scalable and accessible.

Clinically, it is essential to identify if suicidal mental imagery is experienced as comforting, distressing or both. Generally, if a mental activity such as self-generated suicidal mental imagery is perceived as comforting, a person will spend much time in it to escape life’s pain or psych ache. If distressing, a person may try to mentally resist, distract or neutralise it, thus experience great feelings of discomfort. Suicidal mental imagery may start out as having problem-solving capabilities, thereby comforting, but grow to become a mental nuisance when well and wanting to live. This is evidenced in reports of suicidal mental imagery in online platforms, such as Reddit (an online bulletin platform) whereby researchers found that though there was initial relief in suicidal mental imagery as a coping strategy, it often became intrusive, unwanted and the person reporting having lack of control over such imagery (Marchionatti et al., 2024), an inherently deceptive mechanism for coping with life’s pain. Death-related imagery can spontaneously intrude even in the absence of current suicidality, consistent with conditioned cognitive processes and the rapid retrieval of stored representations during times of distress. In CBT terms, this may form part of a vicious cycle linking thoughts, emotions, behaviours, and physiological activation.

This taxonomy may enhance qualitative understanding of injury- and death-related imagery by categorising imagery method (of dying), content, affect (emotional impact), process and appraisals (meanings), referred to here as mechanistic qualities or mechanisms of action. This paper puts forward a taxonomy of first-hand accounts so readers can gain insight into the risk implications of imagery, but also gain an appreciation for why imagery may persist even when well or not actively suicidal. Unpacking individual accounts of imagery may serve as a clinical reference point to aid our understanding of the whole experience of being suicidal and how feelings of associated distress and comfort may act as essential drivers in moving a person from ideation to action. However, in putting forward a taxonomy, authors caution that individualised assessment and treatment of suicidal mental imagery is paramount rather than categorising and making generalisable assumptions of what the outlined images mean for most people.

## 2. Methods

The methodology for the main study is Interpretive Description (ID), a qualitative research design with a central emphasis on producing knowledge that is directly applicable to clinical practice. The meaningful translation of suicidal mental imagery research into practice is a key rationale for selecting ID, as it aligns with the SUMI study objectives and supports the call for frontline services to incorporate suicidal mental imagery assessment into routine mental health care. ID seeks to capture individuals’ subjective experiences with the explicit purpose of using this knowledge to inform and improve practice. The approach employs an inductive analytic process designed to generate clinically relevant understandings of complex phenomena that yield practice-focused implications (Thorne, 2016).

The taxonomy presented in this paper was developed from phase one of a two-phase research study and is based on first-hand accounts of individuals’ lived experiences of suicidal mental imagery. Participants’ experiences were first interpreted inductively to remain grounded in the data, after which the resulting taxonomy was further examined deductively using a priori coding informed by CBT principles. These codes include imagery method, content, affect, process, and appraisal; each is described individually in the results section before the taxonomy is presented.

### 2.1. Ethics

Ethical approval for the study was sought through the Faculty of Health Sciences at Trinity College Dublin and it was approved in April 2023. Given the sensitivity of the study, it was subject to mandatory ongoing Data Protection Office (DPO) oversight and a comprehensive Data Protection Impact Assessment (DPIA) to ensure rigorous risk management. It required the sensitivity of the researcher to ask about imagery experiences, to build rapport and trust early on, to manage changes in emotion during interviews and to respond to these changes appropriately. To support the participant post interview, a list of accessible mental health supports was also developed and provided to each interviewee. Reflexivity following each interview was a critical component of the research process, given the intensity of listening to participants describe detailed and graphic suicidal mental imagery. After each interview, the researcher documented reflections and shared a confidential account of the rich data with the supervisory team.

### 2.2. Sampling

In phase one, purposive sampling was chosen as the preferred sampling framework, a technique widely used in qualitative research in the pursuit of information-rich participants and as the most effective use of limited resources (Patton, 2002). Phase one of the study sought to interview people with specific experience of suicidal mental imagery, as being especially knowledgeable about the research topic of interest. The researcher drew the sample from a national population to include:

1. Adults 18 years and over.
2. An experience of suicidal mental imagery within the last 24 months.
3. Being at least 3 months since last actively suicidal or being out of acute phase, i.e., stabilised.
4. Being located within Ireland.

### 2.3. Recruitment

The study was promoted to individuals in Ireland via social media from September 2023 for an eight-month period. This included more targeted invitations to share the study through voluntary mental health organisation online platforms; however, this approach yielded limited engagement.

Participant recruitment was conducted using a social media flyer featuring a dedicated study logo with a simple design. Participants who met the inclusion criteria volunteered to participate in a semi-structured interview. The interview topic guide was designed in accordance with an established imagery-based cognitive therapy text (Holmes et al., 2019). The interested participant contacted the researcher directly by email or telephone, after which a consent form and participant information leaflet were offered having 7 days to complete consent. At any time, participants could easily withdraw from the study. 16 participants met the inclusion criteria, with one person withdrawing from the study, reporting personal concerns that talking about imagery experiences may impact their mental health wellbeing at that time.

The recruitment flyer and interview guide are provided in the Supplementary Materials.

### 2.4. Data Collection

A total of 15 participants were interviewed, all preferring online or telephone, despite in-person interviews being offered from the outset. Thirteen (*n* = 13) participants requested to meet online through Microsoft Teams or Zoom, and two by telephone. Each interview was between 50 min and 70 min in duration. Each of these was transcribed verbatim and checked multiple times for accuracy. For example, the researcher screened for modulation, nuances in accent, colloquialisms and clarifying meanings while making additional transcript notes highlighting points of concentration in suicidal mental imagery dialogue. In accordance with the DPO and DPIA, all video and audio files were deleted following cross-checking and transcribing. From a gender and age perspective, there was an equal mix of 7 males and 8 females, ranging from 24 to 72 years of age.

Sampling was guided by the concept of ‘information power’ rather than saturation, aiming for a rich interpretation of the interview data. Information power suggests that the more information the sample holds relevant for the actual study, the lower the number of participants that is needed (Malterud et al., 2016). To ensure the study’s credibility, dependability and applicability to practice, the researcher maintained a transparent audit trail, reflexivity post interviews and justifications for how decisions were made, all while maintaining regular supervision. Effective ID research hinges on a dual approach: leveraging the researcher’s clinical expertise (i.e., disciplinary hardwiring) while rigorously mitigating bias to keep the findings grounded in the participant’s perspective. Due to the sensitive nature of the topic, ethical considerations took precedence over formal member-checking. However, four participants followed up after their interviews to share further reflections on their imagery and interview experience, a voluntary addition that greatly enriched the study. All related transcript notes were updated accordingly.

### 2.5. Data Analysis

As mentioned earlier, the taxonomy presented here is drawn from a larger study which sought to explore the real-life experience of suicidal mental imagery from first-hand accounts. Thematic analysis was used to develop three overarching themes, which will be presented in a further paper. Following the articulation of these themes, a further analysis of the data was conducted deductively using a framework informed by the CBT model of treatment. Transcripts were read and re-read, and the descriptions of the imagery were coded and sorted into methods of dying (e.g., cutting or self-stabbing). Following this, data that explained the effect and process associated with the image were also coded and sorted, and an appraisal of image was inductively developed from the first-hand experiences. This was an iterative approach which utilised inductive and deductive reasoning to align each method with the appropriate appraisal until the final taxonomy was complete. A template was designed so that the taxonomy could be presented in summary form. To ensure quality, the first author led the analysis, and regular meetings were held with the research team to discuss the process and ensure that the coding and sorting process fit with the framework, explaining each level of the taxonomy.

## 3. Results

Many participants reported feelings of eagerness alongside surprise when they first encountered the study recruitment call—reflecting both interest and a sense of unexpected recognition. Most participants noted that this was the first time they had seen the concept of ‘suicidal mental imagery’ presented in the public domain and described viewing the invitation as a rare opportunity to speak about their experiences of this imagery, largely for the first time. Thus, the findings from this study indicate that, as it is not normalised to ask about this imagery in clinical practice, it is not volunteered and, as such, consistent with international research findings that are often overlooked. There were feelings of distress and comfort associated with suicidal mental imagery, comforting imagery perceived as more dangerous by study participants. Though the study call was an invitation to the general public, 13 out of the 15 participants volunteered to have a diagnosed major mental illness. These conditions included Bipolar Affective Disorder, Depression, Schizophrenia, Anorexia Nervosa and Borderline Personality Disorder. Participants also reported being regular users of the adult mental health services (both public and private), and over their lifetime, they reported not being asked about suicidal mental imagery experiences. No quantitative data were collected regarding the duration for which participants had experienced this imagery. However, most participants spontaneously reported that they had lived with it since their first experiences of suicidality, often many years earlier, suggesting that it is an enduring aspect of their internal experience. All participants reported that they were engaged in employment or education at the time of the study.

Table 1 outlines the methods of dying represented in participants’ suicidal mental imagery, illustrating how individuals in this study imagined their own death.

All participants in this study had a clear mental picture of the method to die by suicide. Of the 15 participants, two primary methods of dying emerged with equal prominence: 1. cutting or self-stabbing and 2. hanging or choking. Next was overdose or poisoning imagery, and then of equal significance was death by car crash or train tracks and death by drowning. Death by jumping from a height, by self-inflicted gunshot and self-induced starvation were the least likely imagined ways of dying. The discussion will further examine starvation, focusing on its role in triggering suicidal mental imagery in patients with anorexia nervosa.

As mentioned earlier, this taxonomy is categorised into five areas, including imagery method, content, affect, process and appraisal. The method is the way in which people imagine dying by suicide. The content illustrates direct verbatim interview excerpts related to the method of suicide, including contextual information around the suicide act. Affect refers to the emotional tone (distress or comfort) associated with the image. Process refers to the cognitive “rehearsal” function of the image that connects ideation to action—it relates to the structured, cognitive method of breaking down activities such as mental planning into distinct, sequential, and interconnected steps to reach a specific outcome or goal, i.e., death by suicide. The appraisal or ‘meaning making’ refers to subjective cognitive evaluation of suicide (e.g., in ending life’s pain, being at peace) featuring related assumptions or personal significance of death. As the interviews were data-rich, only a sample is illustrated for categorisation in Table 2.

## 4. Discussion

Suicidology research has largely focused on the emergence of suicidal thinking in the context of a mental health condition, problem of living or social determinant (e.g., loneliness or financial strain). Such worthy studies (McClelland et al., 2022; Alothman et al., 2024; Gallagher et al., 2025) may miss vital contextual aspects that in-depth individual accounts can provide on imagery experiences. Quantitative studies can capture some of the content of suicidal or self-harm-based imagery, as demonstrated by Susi et al. (2024) through their employment of online imagery questionnaires; the individual imagery appraisals or meanings may be missed without a strong qualitative arm to such studies. Therefore, an essential purpose of this taxonomy is to encourage suicidal mental imagery dialogue to the fore of assessment, shine a spotlight on suicidal imagery as a serious risk factor for suicide, understand the meaning of certain imagery experiences and also to bridge the research gap from a qualitative perspective.

Suicidal mental imagery research is needed to illuminate the utility of imagery assessment in identifying amplifier risks and steering treatment in relation to the mechanisms of the suicidal mind, and in so doing, direct more targeted interventions. Crane et al. (2012) conceptualised three domains of suicide imagery: image content, associated affect and meaning (appraisal), thereby understanding the mechanisms of what works in mental health care interventions is crucial in stabilisation and lifesaving efforts. For instance, the meaning or appraisal of one person’s self-generated imagery will differ from another person’s imagery, even if they are of similar content. Therefore, because these images are uniquely or exclusively generated by each individual, they require an equally individualised approach to suicidal assessment. Furthermore, a clinician could appraise (on hearing of an exclusive image) that this must be ‘awful’ or distressing for the person, but the person themselves may see it quite differently, for example, appraising the image as being a source of comfort or escape (Crane et al., 2014). Therefore, assessing the content, emotion, and meaning of suicidal imagery is essential, as the way an image is felt and interpreted can drive behaviour. Suicidal imagery is also known to be present at the moments when people are most at risk (Holmes et al., 2007; WeBlau et al., 2015).

Carey and Wells (2019) illustrate a treatment approach utilising imagery assessment through CBT-SP using CBT theory applied to the ‘Flash-Forward’ imagery phenomenon. Flash-forwards were coined by Ng et al. (2016) as a play on ‘flashback’ (traumatic past imagery) and are concerned with future violent daydreaming about suicide, in this instance. Seeing one’s own death ‘in the mind’s eye’ is an emotional and motivational amplifier and is like ‘rocket fuel’ to thoughts, feelings and behaviour. Flash-forwards are a type of mental time travel—the brain receives the imagery information as though it is real and is really about to happen, therefore amplifying the ‘rightness’ or likelihood of an action. This is consistent with emotional amplifier theory as demonstrated in Imagery Enhanced Cognitive Therapy protocols (Holmes et al., 2019). If it is known that imagery acts as an amplifier of thoughts, feelings and associated behaviours, clinicians *need* to ask about it. We know from the available literature that if a clinician does not ask about suicidal mental imagery, it is not volunteered (Holmes et al., 2015; Di Simplicio et al., 2020). Though CBT-SP includes an imagery-based relapse-prevention task in which clients rehearse a past suicidal crisis and practice implementing coping strategies learned during therapy, with the aim of preventing future suicidal crises (Stanley et al., 2009; Wenzel & Jager-Hyman, 2012), such interventions are pitched later in treatment and for a person actively engaged in therapy, often requiring 12+ sessions. While CBT-SP includes imagery-based techniques, suicidal mental imagery is not currently prioritised as a central therapeutic target. Access to CBT-SP and related interventions is also limited, as these approaches typically require referral and are delivered by CBT-trained clinicians who are not commonly based within acute mental health services—settings where individuals often present during suicidal crises. In light of these barriers, it is clinically important to consider opportunities to address suicidal mental imagery proactively within the contexts in which people seek immediate help, through the development of brief, targeted interventions that can be delivered in acute or frontline care environments.

The IMV model (O’Connor & Kirtley, 2018; Cleare & O’Connor, 2026) identifies suicidal mental imagery as a suicide risk in potentially moving a person from ideation to action. This creates a critical gap in stabilisation: a major issue remains unaddressed—unnoticed by clinicians and unspoken by the suicidal person or the ‘elephant in the room’. Clinicians need to start asking about suicidal mental imagery in routine mental health assessments and in understanding the mechanisms of affect, process and appraisal to consider the most appropriate imagery interventions (Carey et al., 2024; Paulik et al., 2023).

Suicidal imagery is, therefore, clinically not a very well-understood experience in practice and an unexplored risk factor for suicidal behaviour. Though models exploring suicide and suicidal behaviour have acknowledged suicidal imagery as a risk factor, for example in CBT, a clinically significant and universally accepted model as endorsed by Rudd (2000) in the cognitive-behavioral model of suicidality and more recently updated in the Integrated Motivational-Volitional Model (IMV) of suicidal behaviour (O’Connor & Kirtley, 2018), it has not been fully translated into routine assessments or interventions. This underscores the need to better understand suicidal mental imagery so we can design prevention strategies that directly target it as a key element of stabilisation. Authors of this manuscript endorse the CBT model as having the most potential in helping demonstrate an imagery intervention for use in practice (Paulik et al., 2023), particularly as it can be adapted from simpler to more advanced applications, dependent on the required needs of the demographic, the service type and at what severity a person may seek help.

The distress and comfort of suicidal mental imagery is an important clinical feature of study findings illustrated in the taxonomy. For instance, distressing imagery in this study presents as an unmet need in the acute hospital environment if clinical staff are unaware of this imagery. To paint a picture, being a patient in a psychiatric ward with intrusive imagery of death by hanging could feel grossly incongruent in the midst of clinical staff simply going about their day, unaware of this phenomenon—it is pain unseen and unasked. This is not a critique of clinical staff in psychiatric settings, but rather a call for meaningful application of suicidal mental imagery research to practice in an area where there is a major lack of knowledge and awareness of the distress associated with imagery. It is hypothesised that the comfort of suicidal imagery is potentially more dangerous as it may have increased capability in ideation to action, albeit a hypothesis requiring further research investigation outside of this study. For instance, there is reference in the transcripts to an appraisal of imagery as a ‘best friend’ in this study, showing that this participant wants to spend time with it, despite having some insight into its dangerousness. Imagery as a self-generated mental activity, it can be hypothesised that spending time in imagery (as a coping strategy for pain/discomfort/escape) can create habituation to pain of death and normality of suicide. The voluntary rehearsal and refinement of suicidal imagery, alongside the relief gained from imaginatively escaping psychological pain, may shape internal evaluations of the mental “rightness” of suicide. Crane et al. (2014) indicate that comfort is associated with several markers of a more severe clinical profile, including both worst-ever prior suicidal ideation and worst suicidal ideation over a 12-month follow-up period. Though more research is needed, the researchers in this paper consider comfort-based imagery as requiring clinical and research attention, a potentially more dominant risk factor in suicidal mental imagery.

God and Jesus came through in a number of participant interviews, but only in one account at the precise point of death. Having a loved one or family member at the point of death was not imagined, but their idiosyncratic presence was typically reflected upon in imagery past the point of death, for instance, at the funeral or imagining talking about the deceased person to another family member. This is experienced as a type of meta-image (having an image about an image) in the same way that we consider meta-cognition (having a thought about a thought). It is the same mental process in that the picture reflects on itself or acts as a representation of another image, a cognitive process where a person is aware of regulating their own thoughts and images. While clinicians may routinely enquire about aspects of suicidal ideation—such as content related to loved ones, plan or intent—the extent to which they explore the presence of an individual’s spiritual beliefs within suicidal mental imagery (e.g., imagery involving God or Jesus, as identified in this taxonomy) is likely to vary and may be influenced by clinician and cultural factors. Therefore, while spirituality is often addressed descriptively within routine mental health assessments, its clinical significance during acute suicidality may be underestimated if it is not explicitly examined through individuals’ suicidal mental imagery, as highlighted in this taxonomy. The authors suggest that opportunities for such content to emerge are increased by directly enquiring about imagery experiences and following that line of exploration, rather than focusing solely on suicidal thoughts, plans, or intent. As demonstrated here, imagery may provide comfort, yet it may also be dangerous if it functions to soothe the individual from ideation toward action. Clinicians therefore need to ascertain whether such imagery is contributing to heightened risk or conversely could be manipulated to act as a protective factor.

Suicidal mental imagery can be experienced in the general population in the absence of a diagnosable mental illness, but imagery can have a more nuanced expression in the presence of a mental illness. For example, in this study, a participant with anorexia nervosa described a slow, starvation-based method of dying expressed through the specific thought patterns of the disorder and imagery focused on the body and its deterioration, i.e., poisoning, sphincter formation and emaciation. It is therefore important to consider the idiosyncratic nature of suicidal mental imagery even further in the context of a particular or enduring mental illness, as having key features of the condition expressed within suicidal mental imagery. This is something that may not be considered by clinicians when assessing suicidality; moreover, the gross absence of critical information when the person is relapsing. The clinician could ask about suicidal mental imagery in the context of the condition, thereby getting a clearer assessment picture in terms of immediate or more longstanding risk factors; clinically, the latter, as it relates to starvation, is not an immediate threat to life, unlike other aforementioned means of suicide in the taxonomy.

## 5. Limitations

The study has some limitations. One limitation was the time gap of up to three months between participants’ most recent experiences of imagery and the interview, which may have affected the accuracy of recall. Ideally, researchers would interview individuals at the point when they are experiencing the most acute suicidal distress (or comfort); however, a prerequisite for participation in this study, due to resources available to researchers and ethical constraints, was that individuals were not actively suicidal at the time of interview. This represents a significant limitation. Despite this requirement, many participants reported that they continue to live with suicidal mental imagery even when they consider themselves to be ‘well’. The findings therefore highlighted the waxing and waning presence of this imagery in participants’ lives rather than its complete absence during periods of improved mental health.

Another potential limitation of the study is the lack of direct engagement with individuals currently engaged in acute mental healthcare services, such as those in inpatient units, home-based treatment or liaison psychiatry services. Though the study attracted people who naturally engage with these services by virtue of having a mental health condition, this study was less targeted by opening it up to the public versus studying a clinical population. However, an alternative interpretation is that the limited open discussion of this phenomenon in Ireland supports the study’s recruitment strategy. The public-facing call for participation, disseminated via social media, may have reached a wider audience (who may or may not have a diagnosable mental illness) and helped validate this experience for individuals who may otherwise have lacked awareness or language to describe it. A key priority of the larger study is to shine a light on suicidal mental imagery in clinical settings but also for the public to be able to speak about it, knowing that this is a real experience for many people despite its unspoken nature.

Despite the richness of the interview data, it is important to emphasise that this taxonomy represents illustrative first-hand accounts from a small sample of 15 individuals native-born to Ireland. Cultural and demographic nuances in suicidal mental imagery experiences are therefore not fully captured in this study. While the overarching premise of the taxonomy remains applicable, the specific content of imagery is likely to reflect place-of-origin–dependent discourse within clinical encounters that explore imagery.

A final limitation of the study relates to the potential for bias, despite efforts to acknowledge and mitigate this risk. The lead author, a cognitive behavioural psychotherapist and mental health nurse researcher, played a central role in developing the CBT codes for this taxonomy, which may have influenced their formulation. To address this, the taxonomy was iteratively reviewed and refined in collaboration with co-authors to ensure accurate categorisation, maintain close alignment with the data, and enhance its clarity, accessibility, and practical utility. Particular attention was given to ensuring the taxonomy was user-friendly for both academic and clinical audiences, with the latter being especially important for the SUMI study in supporting the meaningful translation of findings into practice.

## 6. Recommendations

It is important to consider the challenges clinicians face in fully ascertaining all aspects of suicidality within increasingly brief clinical encounters. While it is essential to ask routine questions regarding suicidal thoughts, plans, and intent, these standard enquiries do not typically capture experiences of suicidal mental imagery. Given time limitations during clinical contacts, clinicians must balance the need to assess a broad range of risk and protective factors with sufficient depth to explore imagery-related experiences, including their content, associated distress or comfort, and personal appraisal. A pragmatic starting point may be to expand standard questioning to include suicidal thoughts and imagery, alongside plan and intent. By briefly explaining what mental imagery is and establishing its relevance for the individual, clinicians can rule it in or out as a key component of risk assessment and intervention. In one-time assessments or first contacts, it is essential at a minimum to establish whether such imagery is present. Normalising imagery as a common aspect of suicidality and conveying that it is amenable to therapeutic intervention may help reduce shame and foster a sense of hope.

As a key recommendation, an essential element is staff training in the assessment and treatment of suicidal mental imagery for practice. Including suicidal mental imagery question prompts in routine mental health assessments is a very important starting point. However, clinicians would also want to know what to do with imagery when it is asked about. Brief imagery intervention training would improve the repertoire of existing suicide prevention skills of qualified staff. The development of a brief CBT-SP Imagery Intervention manual-based approach to assist with this training could help clinicians with the skills and language, but also the confidence in applying suicidal mental imagery for practice. Research is also needed to test imagery interventions and their utility in practice. As a mental health science, increasing the visibility of imagery as a suicide risk factor, the concept needs to be included in university education curricula, specialised suicide prevention training modules and the language of ‘suicidal mental imagery’ in mental health suicide prevention policy documents. Normalising the experience of suicidal mental imagery may therefore be greatly empowered by simply seeing it in documentation. Clinicians may employ brief, imagery-focused interventions as part of acute suicide risk management to support stabilisation, including clarifying intent and planning, addressing access to means, and strengthening protective factors. While the use of imagery-based techniques requires appropriate training and supervision, the development of such skills is generally well within the professional scope of most mental health clinicians, particularly the well-established CBT model of intervention. These interventions can be applied flexibly, ranging from brief, containment-focused work in acute settings to more extended therapeutic interventions within outpatient or community mental health services, when clinically indicated.

Further research is needed to examine both distressing and comfort-based suicidal imagery within clinical populations engaged in mental health services, where suicidal presentations are often prevalent, enduring and have high re-admission or help-seeking. In particular, the hypothesis that some individuals experience suicidal imagery as comforting requires further empirical support through replication studies with a strong qualitative focus. This study is anchored in clinical practice implications, and the identification of comfort-based suicidal imagery underscores a paradoxical risk that warrants careful clinical attention, alongside proactive efforts in assessment and stabilisation.

## 7. Conclusions

Cognitive Behavioural Therapy (CBT) is the preferred evidence-based model in bringing forward suitable suicidal mental imagery interventions for practice. Its utility for practice can be demonstrated in assessment of risk as suicidal mental imagery is uniquely associated with suicide capability (Wetherall et al., 2018; Bayliss et al., 2024); imagery manipulation: image interference to deglamorise the ‘rightness’ or comfort of image (Hales et al., 2011; J. Pearson et al., 2015; Crane et al., 2012; WeBlau et al., 2015; Carey & Wells, 2019); imagery modification: positive goal imagery using Functional Imagery Training (FIT) (Holmes & Mathews, 2005; Di Simplicio et al., 2020) and imagery rescripting or simply put ‘rewriting the story’ (Beck, 1964; Arntz & Weertman, 1999; Arntz, 2012, 2025; Holmes et al., 2015; Paulik et al., 2023).

The method of death in imagery, content of imagery, associated affect, process and appraisal are all mechanistic qualities that may be understood in their separate parts but also as a conceptual understanding (i.e., the whole story) in what this might mean for the suicidal person and what is maintaining suicidal thinking and behaviour. For instance, if we know that comforting imagery seeks to maintain attention by its soothing features, more time is going to be naturally spent daydreaming or fantasising about it, a perceived welcome relief or coping strategy for life’s pain and problems in seeking to escape. Also, what is imagined has sensory qualities, making it feel more ‘real’ (Krüger et al., 2024). If this is the case, clinicians must know about this sensory reality. For example, when one feels comforted, how much time they are spending in this imagery per day/week and if they are indeed consumed by imagery and finding it difficult to consider alternative coping strategies, how habituated they are becoming? If it is a coping strategy, its grip may feel quite strong and compelling. The longer comforting suicidal imagery is engaged with, the more challenging it may be to consider what can equally replace it in terms of soothing affect. Clinicians often employ grounding techniques to help regulate heightened emotions, but it is worth noting that these skilled professionals consider that any suggestions would require satisfactory affect to meet the more habituated comforting aspects of death-based imagery. This is where the skills of the collaborating clinician are important in considering helpful alternatives that the person would want to engage in. Albeit one can only consider these if the mechanisms of suicidal mental imagery are truly understood. If the experience is distressing, where distraction techniques may be helpful or indeed comforting, it can be important to deglamourise the ‘rightness’ of the image, thereby interrupting the rumination. Though some of these interventions may appear ‘difficult to do’ interventions, at first glance, CBT has a unique way of simplifying a concept or mechanism of action more easily through the lens of thoughts, feelings, behaviour and physiological sensations—understandings that most mental healthcare front-facing clinicians have, without being especially core trained as CBT therapists, per se. It is important that suicidal imagery research findings can be applied across a range of mental healthcare professionals. The illustration of a taxonomy informed by key CBT principles may contribute toward this aim.

An intervention can be ‘stepped up’ accordingly, but it is important to meet the needs of the suicidal person where they seek help, at what severity they seek help and that they are cared for suitably by those at the first point of contact (e.g., Primary Care or Emergency Department). For instance, as a general rule of thumb, a person in crisis would need a simple conceptual understanding of why comfort-based suicidal mental imagery could be maintaining suicide risk and what to do to mitigate against this during acuity. For the person, out of crisis, a more detailed or high-level conceptual understanding may be fitting to help manage ongoing chronic suicidal mental imagery. At a basic level, the clinician needs to be able to ask about suicidal mental imagery, provide a brief definition of imagery in lay terms (see Carey et al., 2024), and know what to do at a basic level to ensure the person can speak about their imagery experiences and to identify whether imagery is comforting, distressing or both. Though the taxonomy presented in this paper can go towards these understandings, more research is needed to explore perspectives across clinical and community settings. As a service example, Home-Based Treatment (HBT) mental healthcare offers acute suicidal interventions in the home; therefore, these can be prime services in the application of imagery interventions. While the clinician builds therapeutic rapport, they can employ brief imagery interventions to stabilise risk alongside robust safety planning.

The aim of this research paper was to set out first-hand accounts of people’s experiences of suicidal mental imagery in a research domain where there are limited qualitative studies, despite imagery being a risk factor for suicide. In studies of previously depressed, bipolar, and/or suicidal adults (Crane et al., 2012; Hales et al., 2011; Holmes et al., 2007), 100% of participants reported having experienced suicidal mental imagery at the point they felt most suicidal. Death and injury images act to increase or amplify behaviour (WeBlau et al., 2015; Holmes et al., 2007). In developing a taxonomy of imagery types, authors invite readers and clinicians to view categories of the multifaceted nature of suicidal mental imagery to improve our clinical understanding and apply related interventions to imagery method, content, affect, process and appraisal of imagery. This taxonomy goes towards advancing our understanding of suicidal thinking, highlighting points of heightened risk, and informing more targeted and effective stabilisation efforts in clinical practice. The taxonomy of first-hand accounts seeks to illustrate and understand the experiences of those with death and injury-based images in this study through the lens of CBT and shine a light on application to practice potentials, by unpacking the mechanisms of the suicidal mind. This taxonomy provides a tentative and exploratory insight into the experiences of participants in the larger SUMI study, which may be of relevance to mental health practice. It may assist clinicians’ stabilisation efforts by supporting more nuanced, individualised assessment and treatment of suicidal individuals. By drawing attention to imagery, the taxonomy aligns with and contributes to a developing body of research on suicidal mental imagery, suggesting its potential relevance for inclusion within broader suicide prevention and clinical care frameworks.

## Figures and Tables

**Table 1:** Imagined Methods of Death in Suicidal Mental Imagery.

Method to Die by in Suicidal Mental Imagery	Files 15	References 144
Cutting or self-stabbing	4	14
Hanging or choking	8	13
Overdose or poisoning	5	8
Dying by car crash or on train tracks	4	10
Drowning	4	4
Jumping from a height	2	5
Dying by gunshot	1	2
Starvation	1	5

**Table 2:** A Taxonomy of Suicidal Mental Imagery Types.

Imagery Method	Content	Affect	Process	Appraisal
Cutting or self-stabbing	“You know, in my head I was getting myself to the appointment, and if the appointment didn’t go well, the toilets underneath the office I was going to stab myself to death, and I was going to be found with blood seeping out onto the floor”	Comfort	Voluntary rehearsal and planning to mitigate against unmet needs.	Stabbing self as a quick problem-solving method for managing disappointment. Imagining the appointment with the psychiatrist before it happened and perceiving it to be unhelpful. The clinician would also be shown how unhelpful they were, by witnessing her death by suicide.
Hanging or choking	“Images of like just like hanging from a rope or choking myself with a cable tie…it is like a horror movie in my head on loop, yet the staff tell me turn off the news before bed that it might be distressing. They don’t know what I see”.	Distress	Involuntary intrusive and unwanted imagery; unable to distract from images.	When picturing self-hanging in the wards and getting distressed by it, there is a paradox hearing the night staff ordering a takeaway for their break time. There is a chasm between banal ward activities and horror of distressing suicidal imagery experiences, unable to say anything.
Overdose or poisoning	“So, I would envisage, you know, swallowing caustic soda or sodium hypochlorite, and developing a sphincter, and not being able to eat again… then the other one is always a paracetamol overdose, and in that one I always see myself dying in the hospital, you know where it’s too late. It’s 3 days in, and then I collapse. And then I end up in ICU” “I had very vivid imagery and could mentally see the scenario I would have wanted to do. I went through a number of scenarios and eventually settled on one”.	Comfort	Voluntary; bringing forth familiar self-soothing imagery. long-lasting duration; having rehearsal properties. Grading of imagery types i.e., certain imagery more relieving than others.	By going through the different types of suicidal mental imagery, there is a better chance to find the right one that brings lasting relief. Images that have more detailed extreme endings means higher relief.
Dying by car crash (single car collision)	“Still, sometimes it comes back in, and I do get an of imagine of myself upside down in the field. You know the car in an awful way”. “Yeah, to be like really pulled through the car, like squashed insides—like a human being like a frog, when they are all dissected”.	Both Distress/ Comfort. Ambivalence	Involuntary; ruminating behaviour; thoughts that suicide could eventually happen Voluntary ruminating about work-related stress and unjust actions of others while driving.	If the car crashes suddenly then there will be freedom from all pain. No-one can be annoying or bothersome, if dead. At times, wanting to live and wanting to die in equal measures. Describing self as fat: seeing self all squished and flattened means having another version of the human body—having mastery over body shape, in death.
Drowning self	“Actually, I’m in my car. And I’m going to drive off a pier, and I’m seeing my car floating in water, and me trapped, and me not being able to get out”.	Distress	Involuntary intrusive and unwanted. Punishing self-further.	Feeling trapped in life; seeing own death as awful and suffocating with no way out, imagining pain in death as being a highly traumatic event.
Jumping from a Height	“So, the vision of jumping off a bridge became a very soothing one…the Lord would sort of catch me and bring me up to heaven before I’d hit the ground and lift me up. Yeah”.	Comfort	Voluntary daydreaming, imagery as an avoidance strategy in getting away from day-to-day mundane life tasks. Problem-solving life’s pain.	Being saved from any pain in hitting the ground assuming the Lord catches them—being saved both body and soul.
Dying by gunshot	“I took out my pistol and started killing myself…the imagery I have is always guns”.	Comfort	Voluntary flash imagery of quick, easy mental retrieval. Coping strategy: immediate relief from stress and overwhelm.	Using these mental pictures helps to handle life’s problems.
Dying by self-induced starvation	“Knowing someone who died from anorexia last year, and I saw her in the coffin, and I mean she was very emaciated. I sort of have this image which is like my coffin beside her coffin or also one image of me and her lying in her coffin”.	Comfort	Voluntary soothing fantasy.	Picturing parents speaking to one another over the coffins, saying that the girls are now at complete peace; parents inherently supportive of their suicide. Death = to stop eating = relief to eat = peace.

## Data Availability

There are no publicly archived datasets available due to the sensitive nature of the study. If required, samples of anonymised data can be provided by the principal investigator of the SUMI study to the Editorial Board on request, in accordance with research faculty’s GDPR and DPIA.

## Supplementary Materials

The following supporting information can be downloaded at: https://www.mdpi.com/article/10.3390/bs16060908/s1, (The SUMI study social media flyer; Interview Protocol).
