# Supplementary material for: First-Hand Accounts of Suicidal Mental Imagery: A Taxonomy of Imagery Types
Source: Behav Sci (Basel). 2026 Jun 3;16(6):908. doi: 10.3390/bs16060908 (PMC13295482; doi:10.3390/bs16060908)
Supplement: Supplementary file 1 [file behavsci-16-00908-s001.zip › behavsci-4263005-supplementary.pdf]

Trinity College Dublin
Coláiste na Tríonóide, Baile Átha Cliath
The University of Dublin

The SUMI Study (Marie Carey)

Semi-Structured Interview Introduction

- Commence with a 5-minute meet and greet, discuss interview process and protocol procedures in place.

There will be a direct screening question at the beginning of each interview:

Have you any plans to end your life? Or

Have you been preparing to end your life in any way?

If No:

- Proceed to both a verbalised and written definition of suicidal imagery (screen share written definition, if interviewing using Microsoft Teams, or provide hard copy in person):

Definition of Suicidal Mental Imagery:

What is mental imagery? Mental imagery occurs when perceptual information is accessed from memory, giving rise to the experience of "seeing in the mind's eye" or "hearing in the mind's ear". Images or mental pictures can be both voluntary or involuntary and self-generated. For example, imagine the front door of your house, the windows, the colour and even the sounds as you left home today. You are now creating a mental image. In suicidal mental imagery, people are imagining injury or death to themselves. Sometimes these images give rise to feelings of distress when they are unwanted but also feelings of comfort when they seem to be problem solving. It is common to experience suicidal imagery when suicidal and these images can be short or long lasting, fleeting, still, moving or blurred.

Interview Schedule

1. Can you tell me about a recent time that you were feeling suicidal?

2. When you were feeling down or anxious, was there suicidal imagery that would come into your mind?

   (Prompt: How would the image look if I could see it now?)

3. Have you ever spoken to anyone about your suicidal imagery?

4. Was there more common, repeated or multiple images that came into your mind?

(Prompt: If there are difficulties in describing more rich suicidal imagery, for example, due to memory retrieval or avoidance (as it might be uncomfortable), the researcher can have the participant practice a more neutral image first. For instance, participant taking researcher through the rooms of their house or apartment starting at the front door "Take out your front door key and feel the movement and any sound…walk to the kitchen, open the fridge and bite into a lemon").

5. How vivid or clear was this image? Now let us look at a suicidal image. Can you include as much detail as you can recall, including colours (bright or dull).

   (Prompt: hear, taste, smell, physically feel (senses) and *meaning* or appraisal of the image).

6. From which perspective was the image seen - for example, are you in the picture like watching a film of yourself (i.e. observer perspective) or are you viewing the scene through your own eyes (i.e. field perspective).

7. Is the image like a still picture or a moving film?

8. Do other elements change, for example, other people coming and going in the image?

At this point, the researcher will summarise back to the participant what they have said so far about their more prominent suicidal imagery. This serves two functions: acts as a check that the researcher has understood the participant correctly and it will prompt participant to remember more details.

9. Have you ever felt distressed by suicidal imagery?

If so, can you tell me a little more about that?

10. Have you ever felt comforted or soothed by suicidal imagery?

If so, can you tell me a little more about that?

11. Does the suicidal imagery depend on how you are feeling at the time?

    (Prompt: For example, did you notice when you were feeling distressed that it lasted less or more/ or when you were comforted, did it last less or more). This tests <u>duration</u> of time spent in imagery.

12. Did the suicidal imagery make you want to do anything in response to it and if so, how compelling was this for you?

13. Is there anything else you would like to share about your own experiences of suicidal imagery that we have not covered here today?

Closing remarks thanking participant for their time and re orientation to distress protocol verbally and as screen share written format (mentioned also at the beginning of interview). This protocol will be then directly emailed to each participant at the end of each interview same day. The researcher will reiterate PI contact details email careym2@tcd.ie and telephone 089 2055390.
