# Supplementary material for: First-Hand Accounts of Suicidal Mental Imagery: A Taxonomy of Imagery Types
Source: Behav Sci (Basel). 2026 Jun 3;16(6):908. doi: 10.3390/bs16060908 (PMC13295482; doi:10.3390/bs16060908)
Supplement: Supplementary file 1 [file behavsci-16-00908-s001.zip › The SUMI (Suicidal Mental Imagery) Study.pdf]

The SUMI Study

SUICIDAL MENTAL IMAGERY

TCD research: Mental Imagery in Suicide

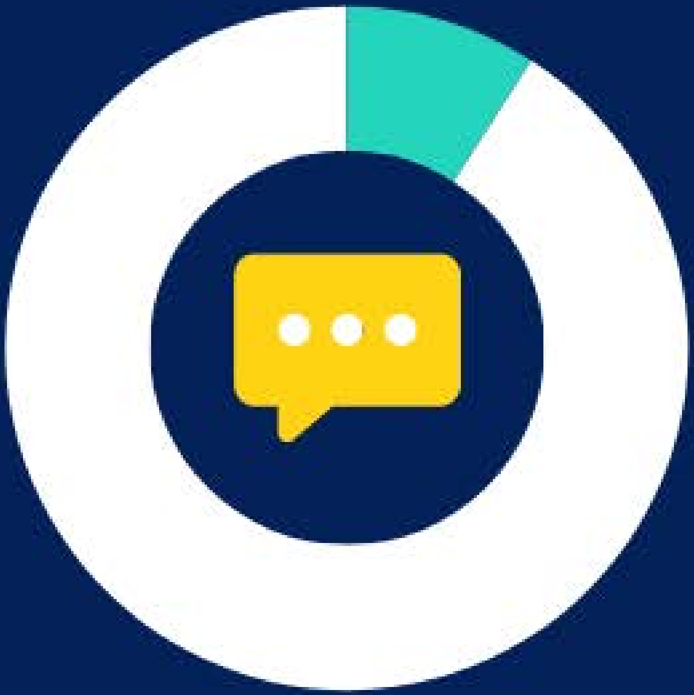

Not all suicidal ideation is experienced through words or thoughts alone

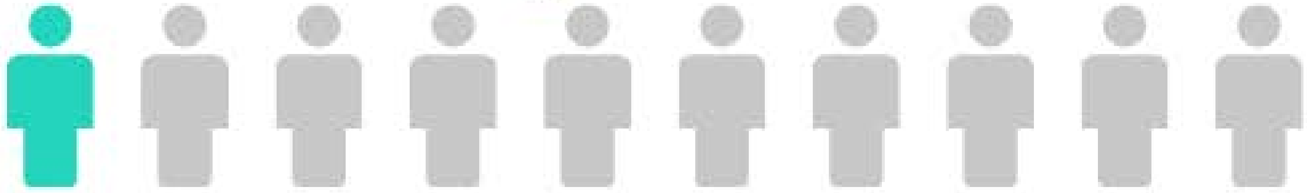

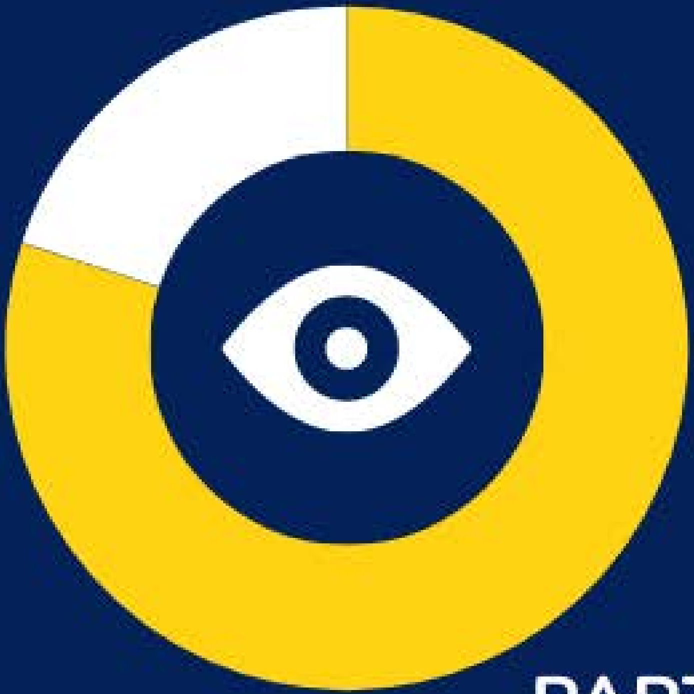

Suicidal imagery or seeing pictures of own injury or death in the 'minds eye' is also common when feeling suicidal

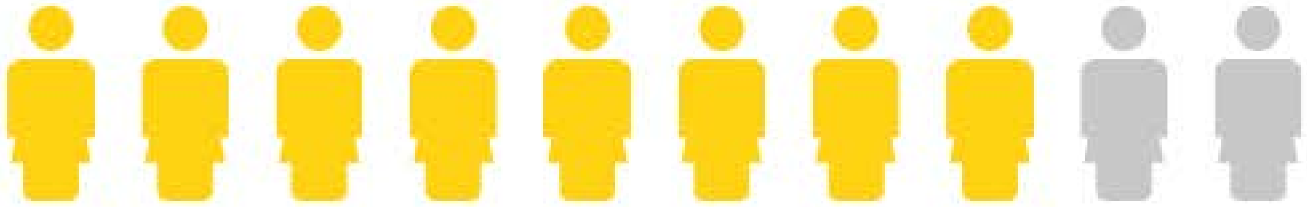

PARTICPANTS NEEDED in Ireland:

Would you be willing to talk to me about your experience of suicidal imagery?

Contact Marie at careym2@tcd.ie or Tel 089 2055390
